# Prevalence and Predictors of Self-Prescribed Vitamin D Supplementation Among University Students in the UAE

**DOI:** 10.3390/nu17182915

**Published:** 2025-09-09

**Authors:** Aaesha H. Alnaqbi, Rubina Sabir, Hafiz M. Shahbaz, Zahra Khan, Mo’ath F. Bataineh

**Affiliations:** 1 Department of Nutrition and Health, College of Medicine and Health Sciences, United Arab Emirates University, Al Ain P.O. Box 15551, United Arab Emirates; 200904873@uaeu.ac.ae (A.H.A.); rubsab@uaeu.ac.ae (R.S.); shahbaz@uaeu.ac.ae (H.M.S.); 2 Nutrition and Health Science, School of Science, University of Greenwich, London ME4 4TB, UK; zahra.khan@greenwich.ac.uk

**Keywords:** self-prescription, dietary supplements, public health, supplement use patterns, health behavior

## Abstract

**Background/Objectives**: Vitamin D deficiency is widespread globally, including in the Middle East. In the UAE, vitamin D deficiency contributes to 78% of bone losses because of cultural and lifestyle factors, which limit sun exposure. Although supplementation is effective, increasing rates of self-prescribed use raise concerns about safety and efficacy, particularly among university students. Therefore, this study aims to assess the prevalence, patterns, and predictors of self-prescribed vitamin D supplementation among university students in the United Arab Emirates. **Methods**: A descriptive cross-sectional survey was conducted among 450 university students aged 18–39 who had used vitamin D supplements in the past 12 months. Data were collected using an online questionnaire and analyzed using chi-square tests and binary logistic regression. **Results**: Among participants, 44.9% reported self-prescribed vitamin D use. Males were more likely to self-prescribe (*p* = 0.010). Self-prescribers used supplements for shorter durations (*p* < 0.001) and were more likely to report motivations like physical performance (*p* = 0.005). Predictors of self-prescription included short-term use (OR = 2.57), non-daily intake (OR = 3.49), use for performance (OR = 2.72), and concurrent vitamin C use (OR = 1.85). **Conclusions**: Self-prescription of vitamin D is common among university students and associated with irregular use and non-clinical motivations, primarily to improve overall health and wellness. While such practices are unlikely to result in toxicity, they may not adequately address the widespread problem of vitamin D deficiency. These findings suggest the need for clear, locally relevant guidance to promote safe and effective supplementation among young adults.

## 1. Introduction

Vitamin D (VD), a fat-soluble prohormone, is essential for calcium–phosphorus metabolism, immune function, and skeletal integrity. Understanding of its role has increased in recent years, with research suggesting potential benefits in modulating chronic disease risks, including cardiovascular, autoimmune, and infectious conditions [1]. Despite this, vitamin D deficiency (VDD) remains a significant global health concern, particularly in regions with low sun exposure or limited dietary intake of VD-rich foods.

Globally, serum concentrations of vitamin D deficiency vary significantly based on geographic location, genetic composition, and population demographics. The International Osteoporosis Foundation (IOF) has found that bone loss is highly prevalent among UAE residents, with a 78% estimated rate of vitamin D deficiency as a contributing factor [2]. Adults in the UAE lack adequate sun exposure even though the UAE is among the sunniest countries in the world, putting adults at higher risk of vitamin D deficiency. This is because dietary and cultural factors like conservative dressing, urban indoor living, and poor food fortification restrict exposure to sunlight [3].

Consequently, VD supplementation has become a common intervention. The UAE, where vitamin D deficiency is highly prevalent, promotes supplementation through clinical guidelines, with higher and risk-stratified doses (400–2000 IU/day) recommended year-round [4].

However, many individuals pursue self-medication, taking VD without a clinical prescription or monitoring based on perceived benefits or informal advice [5]. The practice of using VD preparations for non-specific aches and pains in VD-deficient-endemic regions and over-the-counter VD self-medication has gained prominence [6]. In accordance with research, serum 25-hydroxyvitamin D (25OHD) concentrations higher than 150 ng/mL are toxic levels [7]. Self-prescribed supplementation, labeling errors in VD preparations, or accidental use, such as administration of excessive doses of VD in infants or children for symptoms like delayed teething, “late walking,” and “knock-kneed gait,” have also been reported [3].

This rise in over-the-counter (OTC) supplement use is driven by increased health consciousness, the accessibility of health information online, and the commodification of wellness culture. A study in the UK found that 2.5% of individuals using direct-to-consumer VD testing services had serum 25(OH)D levels exceeding 220 nmol/L, most without medical supervision [5]. Similarly, in Pakistan, a large study analyzing over 100,000 serum samples identified 0.34% with toxic 25(OH)D levels (>150 ng/mL), all of whom were consuming supplements, often for vague symptoms like fatigue or body aches [3].

The risks of excessive, unsupervised VD intake are well-documented. A previous study [8] described adult cases of toxicity linked to non-prescribed supplement regimens leading to renal failure and hospitalization. In Turkey, a study among breastfeeding mothers found that 75.1% used complementary medicine products (CMPs), including VD, primarily to “stay healthy” or “support immunity.” Educational level and being employed were positively associated with CMP use, but, concerningly, 14.4% experienced side effects, and many used these products without professional advice [9]. These findings emphasize how educational and socioeconomic variables shape supplement behaviors and outcomes.

Young adults, particularly university students, are an emerging high-risk group for self-medication. Although typically more educated, they are susceptible to misinformation and peer influence. A study by Chen et al. [10] highlighted that perceived benefits, prior illness, and media were major motivators for VD supplement use. However, knowledge gaps regarding safe dosing and awareness of toxicity risks were widespread. Similar concerns were echoed by Mazhar et al. [11], who found that while awareness of vitamins was high among university students, in-depth knowledge, especially about risks, was lacking.

In addition to health perceptions, the marketing of high-dose VD formulations and bolus regimens contributes to unsafe practices. In Shea et al.’s [5] UK study, users reported taking up to 120,000 IU/day or 300,000–900,000 IU boluses, with only 6.4% under medical oversight. Such dosing exceeds the tolerable upper intake level (UL) of 4000 IU/day recommended by the Institute of Medicine and can precipitate adverse outcomes [5].

These data point to a growing global public health concern. While supplementation is effective in correcting deficiency and preventing disease, improper use can lead to preventable toxicity. This issue is especially pressing in low- and middle-income countries, where regulatory frameworks are weaker, public health education is inconsistent, and healthcare access is variable. To address these challenges, the present study investigates the prevalence, motivations, and associated factors of self-prescribed VD supplementation among university students in the UAE.

## 2. Materials and Methods

### 2.1. Study Design and Participants

This study employed a descriptive cross-sectional design to examine patterns of vitamin D supplementation, with a focus on self-prescribed use among university students. The survey instrument was designed to capture multidimensional data, including demographic characteristics, patterns of supplement consumption, and factors influencing usage behavior. This methodological approach is commonly utilized in public health and nutrition research to assess population-level health behaviors in real-world settings [12].

The sample size was calculated using Raosoft’s online sample size calculator [13]. Based on the total enrolled student population at United Arab Emirates University (UAEU) during the 2022/2023 academic year (*n* = 14,387), with a confidence level of 95%, a margin of error of 5%, and a response distribution of 50%, a minimum of 375 participants was required. Students aged 18 to 39 years who reported using vitamin D supplements within the past 12 months were eligible for inclusion. Of the 685 responses received, 235 were excluded because the respondents reported not using vitamin D supplements in the past 12 months. As the inclusion criteria required vitamin D supplement use, only 450 eligible participants were included in the final analysis. The student cohort was predominantly of Arabian Peninsula origin.

### 2.2. Data Collection Procedure

A structured, self-administered online questionnaire was developed and disseminated via Google Forms (Supplementary Materials). The survey link was distributed to UAEU students through institutional channels, including student mailing lists and campus social media platforms. A convenience sampling technique was employed, facilitating rapid and broad recruitment across different academic disciplines. Data collection occurred between January and April 2023. Ethical approval was obtained from the UAEU Social Sciences Research Ethics Committee (Approval Code: ERS_2019_5989), and informed consent was acquired electronically from all participants prior to survey initiation.

### 2.3. Survey Instrument and Content

The questionnaire was composed of 13 items, grouped into two primary sections. Section A assessed demographic and background characteristics, including age, gender, height, weight, educational level (Bachelor’s, Master’s/Doctorate), academic major (categorized as Medicine/Agriculture, Engineering/Science, Humanities/Social Sciences), and self-perceived health status (poor/fair, good, very good/excellent). Participants were also asked to indicate whether their vitamin D supplementation was prescribed by a healthcare provider or self-initiated.

Section B explored specific patterns and motivations for vitamin D supplement use. This included questions on duration of use, source of supplements (pharmacy, health food store, international sources), reasons for use (e.g., physician-diagnosed deficiency, pregnancy/lactation, beauty enhancement, physical performance, general health maintenance), and label-reading behaviors. An open-ended item allowed participants to list additional supplements used currently or in the past. The questionnaire was adapted from previously validated tools used in studies examining supplement use among university students and general populations [9].

### 2.4. Statistical Analysis

All data were analyzed using IBM SPSS Statistics version 29.0 (IBM Corp., Armonk, NY, USA). Descriptive statistics were used to summarize demographic characteristics and vitamin D usage patterns. Categorical variables were reported as frequencies and percentages, while continuous variables were summarized using means and standard deviations. Normality of distribution was assessed using the Kolmogorov–Smirnov test.

Associations between categorical variables were examined using the chi-square (χ^2^) test. To evaluate predictors of self-prescribed vitamin D use, a binary logistic regression model was constructed with the dependent variable coded as “0” for healthcare-provider-prescribed and “1” for self-prescribed use. Variables were selected for the final multivariate model using univariate logistic regression with a threshold of *p* < 0.20. The final model included adjustments for age and BMI, with adjusted odds ratios (aORs) and 95% confidence intervals (CIs) reported. A *p*-value < 0.05 was considered statistically significant.

## 3. Results

A total of 450 university students met the inclusion criteria, and their data were analyzed in this study. The participants had a mean age of 22.1 ± 4.8 years, and the majority were female (88.4%), with over half (52.2%) aged 21 years or older. Most participants were of normal weight (50.2%), enrolled in undergraduate programs (83.8%), and from Engineering or Science majors (45.1%). Regarding perceived health status, 61.3% rated their health as very good or excellent. Notably, 55.1% reported using vitamin D based on a healthcare provider’s (HCP) prescription, while 44.9% used it through self-prescription (Table 1).

### 3.1. Demographic Factors and Vitamin D Prescription Type

Chi-square analysis revealed a statistically significant association between sex and prescription type (χ^2^ = 6.588, *p* = 0.010), with male participants more likely to self-prescribe vitamin D than females. Other variables, including age group, BMI category, education level, academic major, and self-perceived health status, showed no significant associations with prescription type (*p* > 0.05) (Table 2).

### 3.2. Usage Patterns and Motivations for Vitamin D Supplementation

Participants in the HCP-prescribed group were significantly more likely to use vitamin D on a daily basis (33.1% vs. 13.9%; *p* < 0.001) and for longer durations, including six months or more (33.1% vs. 21.3%; *p* < 0.001). In contrast, those in the self-prescribed group were more likely to report short-term use of one month (37.1% vs. 16.1%).

The most commonly reported reasons for vitamin D supplementation were to improve general health (80.4%) and to treat disease or deficiency (49.1%). Participants using HCP prescriptions were significantly more likely to report disease/deficiency as their primary reason (56.9% vs. 39.6%; *p* < 0.001), whereas self-prescribers more frequently cited improving physical performance (17.3% vs. 8.5%; *p* = 0.005) as a motivating factor. No significant differences were observed between groups in supplement source or instruction-reading habits (*p* > 0.05) (Table 3).

### 3.3. Co-Use of Other Dietary Supplements

**Figure 1:**
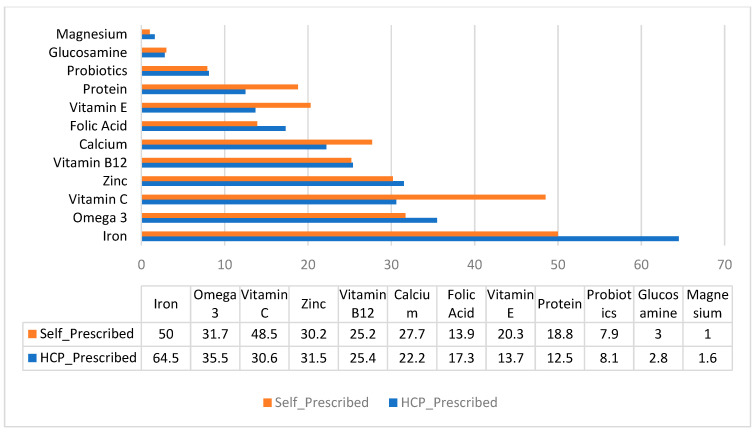
Percentage of vitamin D users who ingested other dietary supplement products categorized by prescription type. The bars do not add up to 100% due to the selection of multiple answers.

The majority of vitamin D users also reported taking additional dietary supplements (91.9% HCP-prescribed vs. 86.6% self-prescribed; *p* = 0.067). The average number of other supplements used did not differ significantly between groups (3.0 ± 2.4 vs. 3.2 ± 2.5; *p* = 0.378). Significant associations were found between vitamin D prescription type and the use of iron (χ^2^ = 9.630, *p* = 0.002) and vitamin C (χ^2^ = 14.990, *p* < 0.001), with iron being more common among HCP prescribers and vitamin C among self-prescribers (Figure 1).

### 3.4. Predictors of Self-Prescribed Vitamin D Use

Binary logistic regression identified several significant predictors of self-prescribed vitamin D use. Participants reporting shorter durations of supplement use were more likely to self-prescribe, particularly those using supplements for 1 month (reference group) compared to 2–5 months or 6 months or more (*p* = 0.001). Similarly, non-daily supplement users were more likely to self-prescribe (OR = 0.287, 95% CI: 0.156–0.526, *p* < 0.001). The use of vitamin D to enhance physical performance was a significant predictor (OR = 2.724, 95% CI: 1.276–5.818, *p* = 0.010), as was co-use of vitamin C (OR = 1.850, 95% CI: 1.134–3.019, *p* = 0.014). Other demographic or supplement-related factors did not reach statistical significance (Table 4).

## 4. Discussion

This study investigated the prevalence and determinants of self-prescribed vitamin D supplement use among university students in the UAE. According to research, the UAE has a high level of VDD among young adult Emirati University students (mean serum 25-hydroxyvitamin D level 20.9 nmol/L in females vs. 27.3 nmol/L in males) in relation to sun avoidance behaviors [2]. Previous research conducted at UAE University revealed that over 70% of female students did not consume sufficient vitamin D through diet, and 37% (40% of dorm residents) were considered insufficient (intake < 5 µg/day) [14]. In Jordan, a comparable study at Hashemite University reported that 31.2% of female students were vitamin D deficient. Limited daily sun exposure (≥30 min) was associated with more than fourfold higher odds of deficiency [15].

The results of this study revealed that nearly 45% of vitamin D users had not received medical guidance, highlighting a significant prevalence of self-medication among young adults. This finding aligns with previous studies from various regions, including the UK, Pakistan, and Turkey, where university students exhibited high rates of unsupervised supplement use, often driven by perceptions of safety and general health improvement [3,5,9]. A study from South Africa found that 42% of university students regularly used dietary supplements, with vitamin–mineral combinations [16].

Similarly, a recent cross-sectional study from Tikrit University by [17] identified vitamin D as one of the most self-prescribed supplements among students, alongside vitamin C and omega-3, with most participants citing health maintenance or immune boosting as their motivation.

This research revealed that male students were more inclined than their female counterparts to self-prescribe vitamin D, even though the latter represented the majority of the respondents. The gender imbalance in our sample, with fewer male respondents, may reflect both the higher proportion of female students enrolled in health-related disciplines and potentially lower engagement of males with vitamin D supplementation, as suggested by previous research [18]; this may have influenced the observed gender-specific patterns.

This finding aligns with patterns observed in previous research by [16], suggesting that males may be more inclined toward self-directed health behaviors, including supplement use without professional guidance. A significant association was found between overall awareness of vitamin D and the intake of at least two sources of the nutrient among all participants, with higher intake observed among those who were aware (45.0% vs. 33.9%, *p* = 0.012). When stratified by gender, this relationship was markedly stronger and statistically significant in males (57.3% vs. 33.3%, *p* < 0.001) but nonsignificant among females (34.6% vs. 35.4%, *p* = 0.920) [19]. In contrast, the study by Tariq et al. [18] indicated that supplement usage was significantly higher in female students (F = 52% vs. M = 37%; *p* = 0.003). This gender-specific deviation highlights the need for gender-specific health education programs that deal with both overuse of self-medicating habits by men and evidence-based supplement use by women.

Self-prescribing individuals are more inclined to take vitamin D for shorter durations and for non-clinical deficiency purposes, such as performance enhancement. This behavioral pattern is consistent with findings from [8], who reported that vitamin D was commonly used for general health benefits by active adults owing to fitness culture and commercial marketing. Despite non-conclusive evidence, people have been misled that taking excessive doses of vitamin D may result in miraculous results [8].

As a consequence, the demand for its supplementation has escalated either through doctors’ or nutritionists’ prescriptions or even by regular media communications without scientific backing [8]. Similarly, Ficarra et al. [20] reported that sports science students often used vitamin D and other supplements without medical consultation, motivated more by physical training demands than health indicators.

In contrast, those using vitamin D under healthcare supervision reported longer durations and daily intake, supporting structured and evidence-based use, as also shown by findings from [21], who conducted a randomized controlled trial in Pakistan showing high compliance and optimal outcomes among participants receiving supervised high-dose vitamin D supplementation in clinical settings. These findings reiterate the importance of medical supervision to ensure proper dosing, safe monitoring, and efficacy of vitamin D use, especially because self-directed supplementation is on the rise due to misinformation and the wellness industry.

Notably, 80.4% of participants cited general health improvement as the reason for taking vitamin D, while nearly half cited illness or deficiency. However, only 56.9% of users prescribed vitamin D by an HCP claimed they were aware of such a deficiency, indicating that even clinically guided usage may lack biochemical evidence. This aligns with findings by [5], who reported widespread use of high-dose vitamin D supplements among users without documented deficiency, many exceeding safe upper intake limits. Similarly, the consensus by [22] stresses that supplementation should follow documented need, noting the potential for toxicity and unnecessary healthcare costs when vitamin D is used outside of clear clinical indications. Moreover, Ogorek & Samara [23] discussed the emerging shift in clinical practice, with some healthcare systems limiting routine population-level vitamin D testing due to overuse and misuse.

Co-supplementation with other vitamins and minerals was also widespread, with vitamin C and iron reported as the most common. There was a higher incidence of self-prescribers reportedly taking vitamin C along with vitamin D. This behavior has been more prominent in post-COVID-19 studies about immune support [24]. Iron, on the other hand, was more common among HCP-prescribed users, likely reflecting clinical indications, such as anemia or pregnancy-related deficiencies [9]. Although multi-supplement use is not inherently harmful, unsupervised combinations raise the risk of nutrient–nutrient interactions, particularly in fat-soluble vitamins like D, where toxicity is cumulative [8,25,26]. Furthermore, the concurrent use of vitamin D with other antioxidants, such as omega-3 fatty acids and vitamin E, popular in fitness and self-medication circles, has been documented to be driven more by market influence than clinical necessity [24].

The logistic regression model identified four significant predictors of self-prescription: shorter duration of use, non-daily intake, use for physical performance, and concurrent vitamin C supplementation. These predictors point toward sporadic, lifestyle-oriented usage rather than structured medical management. A recent cross-sectional study conducted among university students in Jordan by [27] found that over 60.9% of participants reported using dietary supplements (DS), most commonly single-nutrient formulas like vitamin D, vitamin C, and iron. Despite high usage rates, participants displayed poor knowledge and high-risk practices, particularly among those driven by general wellness motives rather than clinical indications. The majority cited health maintenance rather than deficiency correction as their motivation, and supplement use was significantly higher among undergraduates and those with low to middle incomes, highlighting the sociocultural reach of unregulated supplement markets [27]. These findings align with our results, where self-prescribed users engaged in co-supplementation and relied on perceived rather than tested need. Such behaviors have been associated with poor label adherence and heightened risk of overuse or improper dosing [3,5].

From a public health perspective, the normalization of self-medication with vitamin D, especially in student populations, warrants attention. Although awareness of vitamin D’s health benefits has grown, studies show persistent gaps in knowledge regarding dosing, toxicity thresholds, and appropriate indications [28,29]. Educational interventions are needed to improve supplement literacy and promote safer, evidence-based decision making, particularly in university health services. While the UAE has published clinical practice guidelines recommending risk-stratified daily supplementation (400–2000 IU depending on age, pregnancy, or risk factors) [4], these recommendations are mainly disseminated through healthcare providers rather than broad public health campaigns. In contrast, countries like the UK provide population-wide messages that clearly specify standard daily intakes for the general population and additional advice for pregnancy and lactation. This highlights the need for the UAE to complement existing clinical guidelines with government-led public health messaging to ensure consistency, accessibility, and uptake across the wider population.

Finally, although cases of vitamin D toxicity remain rare, reports like those by [8,25] illustrate the potential for life-threatening hypercalcemia and renal impairment with chronic high-dose use. In our study, self-prescribed users were more likely to engage in such patterns of unsupervised, intermittent use, underscoring the importance of regulatory and clinical oversight even in seemingly low-risk populations. However, this pattern is still concerning if individuals are vitamin D deficient, as irregular and unsupervised intake may prevent correction of deficiency while giving a false sense of adequacy. This underscores the importance of both regulatory oversight and educational strategies to encourage safe, consistent, and evidence-based supplementation.

Limitations of this study include the use of convenience sampling and reliance on self-reported data, which may introduce recall and selection bias. Moreover, the use of convenience sampling resulted in the under-representation of male students, which may have skewed the findings. Another limitation of this study is that the survey did not collect data on the average dosage of vitamin D consumed. This was beyond the original scope of the project, and therefore dosage-specific patterns could not be analyzed. Additionally, serum 25(OH)D levels were not measured, limiting clinical correlation. Nonetheless, the study provides important insights into the behavioral trends and determinants of vitamin D use in a previously under-researched population.

## 5. Conclusions

This study highlights the high prevalence of self-prescribed vitamin D supplementation among university students in the UAE. Nearly half of the participants reported using vitamin D without medical consultation, most often in short-term and irregular ways motivated by general health or performance benefits. While such practices are unlikely to result in toxicity, they may not adequately address the widespread problem of vitamin D deficiency. These findings underscore the importance of clear, locally relevant public health guidance and educational initiatives to promote safe and effective supplementation practices. Further research, including biochemical assessments, is needed to determine whether current self-directed use sufficiently improves vitamin D status in this population.

## Figures and Tables

**Table 1:** Characteristics of study participants (*n* = 450).

Variable	Value
Sex, *n* (%)	
Female	398 (88.4)
Male	52 (11.6)
Age (years), mean (SD)	22.1 (4.8)
Age, *n* (%)	
<21	215 (47.8)
≥21	235 (52.2)
Weight (kg), mean (SD)	61.9 (15.6)
Height (cm), mean (SD)	161.0 (10.0)
BMI (kg/m^2^), mean (SD)	23.8 (5.2)
BMI, *n* (%)	
Underweight	69 (15.33)
Normal	226 (50.22)
Overweight	95 (21.11)
Obesity	60 (13.33)
Education, *n* (%)	
Bachelor	377 (83.8)
Master/Doctorate	73 (16.2)
Academic major, *n* (%)	
Engineering/Science	203 (45.11)
Humanities/Social Sciences	137 (30.44)
Medicine/Agriculture	110 (24.44)
Self-perceived health, *n* (%)	
Poor/fair	48 (10.7)
Good	126 (28.0)
Very good/excellent	276 (61.3)
Vitamin D prescription type, *n* (%)	
Self-prescribed	202 (44.9)
HCP-prescribed	248 (55.1)

**Table 2:** Association of demographic factors with vitamin D supplement prescription type (*n* = 450).

Variable	Prescription Type		χ^2^	*p*-Value *
	HCP 248 (55.1%)	Self 202 (44.9%)		
Sex, *n* (%)			6.588	0.010
Female	228 (91.9)	170 (84.2)		
Male	20 (8.1)	32 (15.8)		
Age, *n* (%)			0.227	0.634
<21	121 (48.8)	94 (46.5)		
≥21	127 (51.2)	108 (53.5)		
BMI, *n* (%)			4.608	0.203
Underweight	46 (18.5)	23 (11.4)		
Normal	122 (49.2)	104 (51.5)		
Overweight	49 (19.8)	46 (22.8)		
Obesity	31 (12.5)	29 (14.4)		
Self-perceived health, *n* (%)			1.994	0.369
Poor/fair	31 (12.5)	17 (8.4)		
Good	69 (27.8)	57 (28.2)		
Very good/excellent	148 (59.7)	128 (63.4)		
Education, *n* (%)			0.004	0.953
Bachelor	208 (83.9)	169 (83.7)		
Master/Doctorate	40 (16.1)	33 (16.3)		
Academic major, *n* (%)			3.390	0.184
Engineering/Science	115 (46.4)	88 (43.5)		
Humanities/Social Sciences	67 (27.0)	70 (34.7)		
Medicine/Agriculture	66 (26.6)	44 (21.8)		

HCP: healthcare provider. * Based on chi-square analysis

**Table 3:** Usage duration, pattern, reasons, and sources of vitamin D supplements according to prescription type (*n* = 450).

Variable	Prescription Type		χ^2^	*p*-Value *
	HCP 248 (55.1%)	Self 202 (44.9%)		
Duration of use, *n* (%)			26.118	<0.001
1 month	40 (16.1)	64 (37.1)		
2 months	67 (27.0)	69 (34.2)		
3–5 months	59 (23.8)	26 (12.9)		
6 months or more	82 (33.1)	43 (21.3)		
Daily supplement use, *n* (%)	82 (33.1)	28 (13.9)	22.227	<0.001
Source of used supplement, *n* (%)			8.763	0.067
Pharmacy/over the counter	77 (31.0)	78 (38.6)		
Healthy food store	76 (30.6)	55 (27.2)		
Foreign country	15 (6.0)	11 (5.4)		
Other	50 (20.2)	24 (11.9)		
Do not know	30 (12.1)	34 (16.8)		
Reason/s for supplement use, *n* (%)				
Disease/deficiency	141 (56.9)	80 (39.6)	13.256	<0.001
Pregnancy/lactation	18 (7.3)	22 (10.9)	1.814	0.178
Beauty	77 (31.0)	69 (34.2)	0.491	0.483
Improve health	192 (77.4)	170 (84.2)	3.214	0.073
Improve physical performance	21 (8.5)	35 (17.3)	8.019	0.005
Always read instructions, *n* (%)	128 (51.6)	100 (49.5)	0.198	0.656

HCP: healthcare provider. * Based on chi-square analysis

**Table 4:** Relationship between demographic factors, pattern of supplement usage and other additional supplement products, and self-prescribed vitamin D use (*n* = 450).

Variable	Odds Ratio	95% CI		*p*-Value
		Lower	Upper	
Sex				0.220
Female	1			
Male	1.556	0.767	3.155	
Academic major				0.176
Engineering/Science	1			
Humanities/Social Sciences	1.608	0.976	2.649	
Medicine/Agriculture	1.224	0.712	2.105	
Duration of use				0.001
1 month	1			
2 months	0.512	0.284	0.922	
3–5 months	0.280	0.139	0.567	
6 months or more	0.318	0.166	0.611	
Daily supplement use				<0.001
No	1			
Yes	0.287	0.156	0.526	
Source of used supplement				0.155
Pharmacy/over the counter	1			
Healthy food store	1.710	0.991	2.950	
Foreign country	1.323	0.494	3.544	
Other	0.799	0.405	1.576	
Do not know	1.393	0.696	2.784	
Diseases/deficiency				0.080
No	1			
Yes	0.673	0.432	1.049	
Pregnancy/lactation				0.860
No	1			
Yes	0.929	0.412	2.095	
Improve health				0.388
No	1			
Yes	1.287	0.726	2.281	
Improve physical performance				0.010
No	1			
Yes	2.724	1.276	5.818	
Vitamin C				0.014
No	1			
Yes	1.850	1.134	3.019	
Vitamin E				0.412
No	1			
Yes	1.305	0.691	2.467	
Calcium				0.276
No	1			
Yes	1.369	0.778	2.410	
Iron				0.061
No	1			
Yes	0.656	0.422	1.020	
Protein				0.825
No	1			
Yes	1.081	0.542	2.156	

Adjusted for age and BMI.

## Data Availability

The data presented in this study are available on request from the corresponding author due to ethical reasons.

## Supplementary Materials

The following supporting information can be downloaded at https://www.mdpi.com/article/10.3390/nu17182915/s1. File S1: Prevalence and Predictors of Self-Prescribed Vitamin D Supplementation Among University Students in the UAE.

## References

[1] Izzo M., Carrizzo A., Izzo C., Cappello E., Cecere D., Ciccarelli M., Iannece P., Damato A., Vecchione C., Pompeo F. (2021). Vitamin D: Not just bone metabolism but a key player in cardiovascular diseases. Life.

[2] Salmanpour V.A., Ibrahim H.S., Salameh A.G., Yahya A.M., Debal B.K. (2016). Vitamin D deficiency: Knowledge and practices among the adult population in Sharjah, United Arab Emirates. Arch. Osteoporos..

[3] Muneer S., Siddiqui I., Majid H., Zehra N., Jafri L., Khan A.H. (2022). Practices of vitamin D supplementation leading to vitamin D toxicity: Experience from a low-middle income country. Ann. Med. Surg..

[4] Haq A., Wimalawansa S.J., Pludowski P., Al Anouti F. (2018). Clinical practice guidelines for vitamin D in the United Arab Emirates. J. Steroid Biochem. Mol. Biol..

[5] Shea R.L., Berg J.D. (2017). Self-administration of vitamin D supplements in the general public may be associated with high 25-hydroxyvitamin D concentrations. Ann. Clin. Biochem..

[6] Taylor P.N., Davies J.S. (2018). A review of the growing risk of vitamin D toxicity from inappropriate practice. Br. J. Clin. Pharmacol..

[7] Fedorov Y., Kovpak A. (2025). Vitamin D Toxicity and Clinical Consequences of Hypervitaminosis. SSP Mod. Pharm. Med..

[8] de Paula A.L.T., Gonzaga W.P.F., Oliveira L.M., Feibelmann T.C.M., Markus J. (2020). Exogenous intoxication by non-prescribed use of vitamin D, a case report. BMC Geriatr..

[9] Bilgin D.D., Karabayir N., Çetinkaya H.B., Kacir A., Öçal Ö., Başibüyük M., Büke Ö. (2025). Reasons, associated factors, and attitudes toward breastfeeding mothers’ use of complementary medicine products: A study from Türkiye. Int. Breastfeed. J..

[10] Chen Y.-H., Chao S.-L., Chu Y.-W. (2022). Effects of perceived benefit on vitamin D supplementation intention: A theory of planned behaviour perspective. Int. J. Environ. Res. Public Health.

[11] Mazhar I., Rai M.M., Khan Q.U., Pramaningtyas M.D., Aaien K.U., Zia R. (2023). Determining Awareness of Vitamin D Necessity, Deficiency and its Appropriate Attainment among Medical undergraduate students of a private medical college: A Descriptive cross-sectional study. Pak. J. Med. Health Sci..

[12] Al Hadhrami R.S., Al Kaabi R., Al Shuaibi H.J., Al Abdulsalam R.S. (2024). Assessment of vitamin D-related knowledge, attitudes and practices among Sultan Qaboos University students in Oman: A cross-sectional study. BMJ Public Health.

[13] Raosoft Inc Sample Size Calculator. http://www.raosoft.com/samplesize.html.

[14] Laleye L.C., Kerkadi A.H., Wasesa A.A., Rao M.V., Aboubacar A. (2011). Assessment of vitamin D and vitamin A intake by female students at the United Arab Emirates University based on self-reported dietary and selected fortified food consumption. Int. J. Food Sci. Nutr..

[15] Qatatsheh A., Tayyem R., Al-Shami I., Al-Holy M.A., Al-Rethaia A.S. (2015). Vitamin D deficiency among Jordanian university students and employees. Nutr. Food Sci..

[16] Steele M., Senekal M. (2005). Dietary supplement use and associated factors among university students. S. Afr. J. Clin. Nutr..

[17] Noaman A.M. (2024). Study the Prevalence of Dietary Supplements Use Among Students in Tikrit University. Coll. Lit..

[18] Tariq A., Khan S.R., Basharat A. (2020). Assessment of knowledge, attitudes and practice towards Vitamin D among university students in Pakistan. BMC Public Health.

[19] Alharbi A.A., Alharbi M.A., Aljafen A.S., Aljuhani A.M., Almarshad A.I., Alomair I.A., Alfalah M.A. (2018). Gender-specific differences in the awareness and intake of Vitamin D among adult population in Qassim Region. J. Fam. Community Med..

[20] Ficarra G., Rottura M., Irrera P., Bitto A., Trimarchi F., Di Mauro D. (2022). Use of drugs and dietary supplements in university students of sports science: Results of a survey-based cross-sectional study. Nutrients.

[21] Saleem J., Zakar R., Butt M.S., Kaleem R., Chaudhary A., Chandna J., Jolliffe D.A., Piper J., Abbas Z., Tang J.C. (2025). High-dose vitamin D3 to improve outcomes in the convalescent phase of complicated severe acute malnutrition in Pakistan: A double-blind randomised controlled trial (ViDiSAM). Nat. Commun..

[22] Giustina A., Bilezikian J.P., Adler R.A., Banfi G., Bikle D.D., Binkley N.C., Bollerslev J., Bouillon R., Brandi M.L., Casanueva F.F. (2024). Consensus statement on vitamin D status assessment and supplementation: Whys, whens, and hows. Endocr. Rev..

[23] Ogorek A.N., Samara V.A. (2025). New Clinical Practice Guidelines for Vitamin D Supplementation and Testing. Clin. Chem..

[24] Aversa R., Petrescu R.V., Apicella A., Petrescu F.I. (2016). We are addicted to vitamins C and EA review. Am. J. Eng. Appl. Sci..

[25] Virú-Loza M.A., Alvarado-Gamarra G., Zapata-Sequeiros R.I., Flores-Nakandakare H.F. (2024). Life-threatening hypercalcemia in a child with vitamin D intoxication due to parental self-medication: A case report. SAGE Open Med. Case Rep..

[26] Maggini V., Crescioli G., Ippoliti I., Gallo E., Menniti-Ippolito F., Chiaravalloti A., Mascherini V., Da Cas R., Potenza S., Gritti G. (2023). Safety profile of vitamin D in Italy: An analysis of spontaneous reports of adverse reactions related to drugs and food supplements. J. Clin. Med..

[27] Elsahoryi N.A., Odeh M.M., Jadayil S.A., McGrattan A.M., Hammad F.J., Al-Maseimi O.D., Alzoubi K.H. (2023). Prevalence of dietary supplement use and knowledge, attitudes, practice (KAP) and associated factors in student population: A cross-sectional study. Heliyon.

[28] Dominguez L.J., Farruggia M., Veronese N., Barbagallo M. (2021). Vitamin D sources, metabolism, and deficiency: Available compounds and guidelines for its treatment. Metabolites.

[29] Zadka K., Pałkowska-Goździk E., Rosołowska-Huszcz D. (2018). The state of knowledge about nutrition sources of vitamin D, its role in the human body, and necessity of supplementation among parents in central Poland. Int. J. Environ. Res. Public Health.

