# Supplementary material for: Prevalence and Predictors of Self-Prescribed Vitamin D Supplementation Among University Students in the UAE
Source: Nutrients. 2025 Sep 9;17(18):2915. doi: 10.3390/nu17182915 (PMC12472534; doi:10.3390/nu17182915)
Supplement: Supplementary file 1 [file nutrients-17-02915-s001.zip › nutrients-3845018-supplementary.pdf]

Prevalence and Predictors of Self-Prescribed Vitamin D Supplementation Among University Students in the UAE

Section A: Demographic Information

1. Sex:
a) Female
b) Male

2. Age (years): ___________

3. Weight (kg): ___________

4. Height (cm): ___________

5. Level of Education:
a) Bachelor's
b) Master's/Doctorate

6. Academic Major:
a) Engineering/Science
b) Humanities/Social Sciences
c) Medicine/Agriculture

7. Self-Perceived Health Status:
a) Poor/Fair
b) Good
c) Very Good/Excellent

8. Type of Vitamin D Supplementation:
a) Self-prescribed
b) Prescribed by a healthcare professional (HCP)

Section B: Vitamin D Supplement Use Patterns

9. Duration of Vitamin D Supplement Use:
a) 1 month
b) 2 months
c) 3–5 months
d) 6 months or more

10. Source of the Vitamin D Supplement:
a) Pharmacy/Over-the-counter
b) Health food store
c) Purchased from a foreign country
d) Other (please specify): ___________
e) Don't know

11. Primary Reason(s) for Using Vitamin D Supplements (check all that apply):
☐ Disease/Deficiency
☐ Pregnancy/Lactation
☐ Beauty-related reasons
☐ To improve general health
☐ To enhance physical performance

12. How often do you read and follow the instructions on the supplement label?
a) Never
b) Sometimes
c) Always

13. Which dietary supplements do you currently use or have used?
Please list all that apply:

____
